# Enhancement of acetate production in hydrogen-mediated microbial electrosynthesis reactors by addition of silica nanoparticles

**DOI:** 10.1186/s40643-023-00627-6

**Published:** 2023-01-20

**Authors:** Zeyan Pan, Zhuangzhuang Liu, Xiaona Hu, Kai Cui, Wenfang Cai, Kun Guo

**Affiliations:** 1 School of Chemical Engineering and Technology, Xi’an Jiaotong University, Xi’an, 710049 China; 2 College of Veterinary Medicine, Northwest A&F University, Yangling, 712100 China; 3 School of Ecology and Environment, Zhengzhou University, Zhengzhou, 450001 China

**Keywords:** Microbial electrosynthesis, H_2_-mediated, Silica nanoparticles, Mass transfer, Acetate production

## Abstract

Microbial electrosynthesis (MES) is a promising technology for CO_2_ fixation and electrical energy storage. Currently, the low current density of MES limits its practical application. The H_2_-mediated and non-biofilm-driven MES could work under higher current density, but it is difficult to achieve high coulombic efficiency (CE) due to low H_2_ solubility and poor mass transfer. Here, we proposed to enhance the hydrogen mass transfer by adding silica nanoparticles to the reactor. At pH 7, 35 ℃ and 39 A·m^− 2^ current density, with the addition of 0.3wt% silica nanoparticles, the volumetric mass transfer coefficient (*k*_La_) of H_2_ in the reactor increased by 32.4% (from 0.37 h^− 1^ to 0.49 h^− 1^), thereby increasing the acetate production rate and CE of the reactor by 69.8% and 69.2%, respectively. The titer of acetate in the reactor with silica nanoparticles (18.5 g·L^− 1^) was 56.9% higher than that of the reactor without silica nanoparticles (11.8 g·L^− 1^). Moreover, the average acetate production rate of the reactor with silica nanoparticles was up to 2.14 g·L^− 1^·d^− 1^ in the stable increment phase, which was much higher than the other reported reactors. These results demonstrated that the addition of silica nanoparticles is an effective approach to enhancing the performance of H_2_-mediated MES reactors.

**Graphical Abstract:**

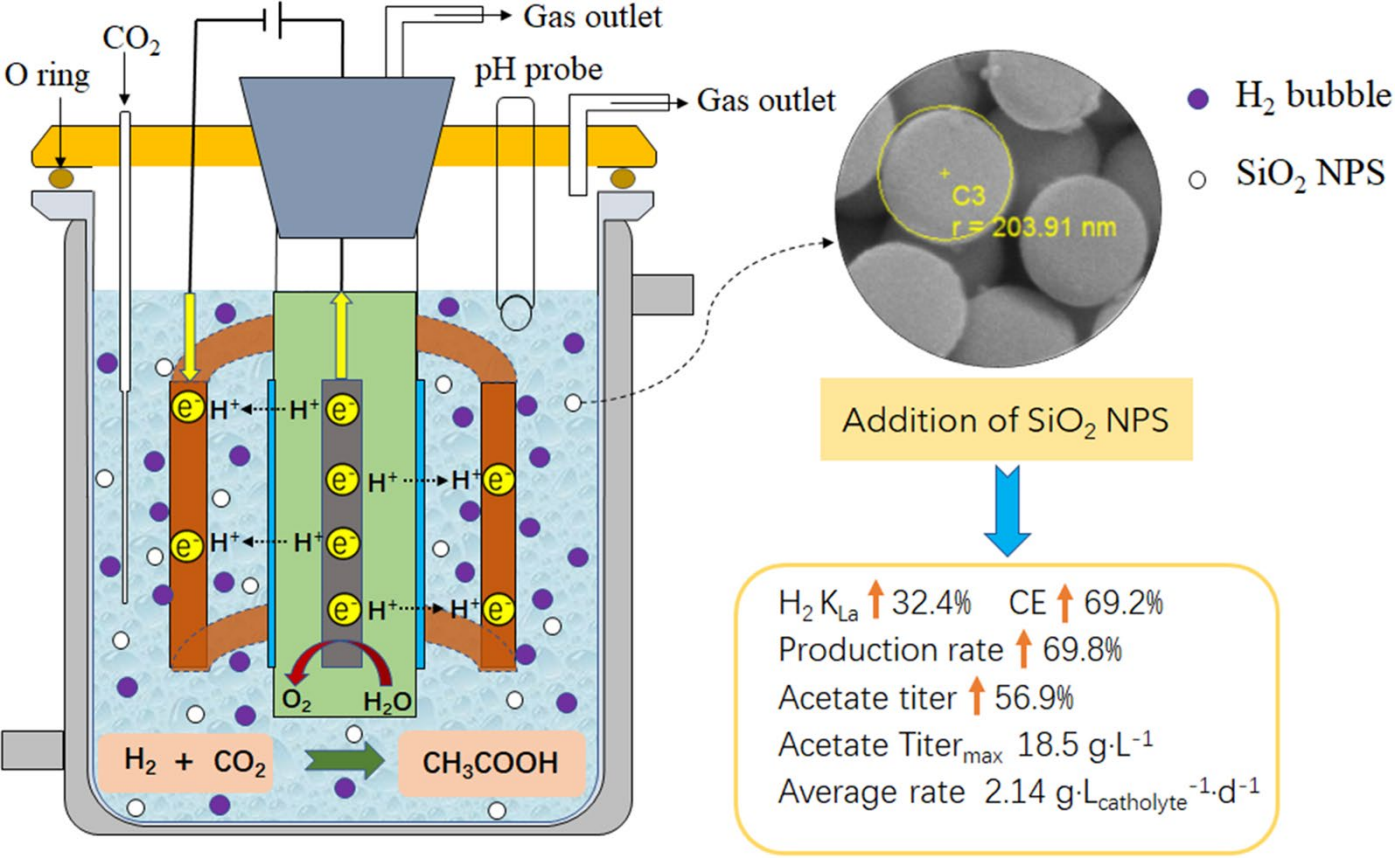

**Supplementary Information:**

The online version contains supplementary material available at 10.1186/s40643-023-00627-6.

## Introduction

Microbial electrosynthesis (MES) is a process that utilizes microorganisms as the cathodic catalysts for electrochemical CO_2_ reduction (Nevin et al. 2011). Compared to abiotic catalysts, microorganisms have the advantages of high selectivity, self-regenerating ability, and capability of producing multi-carbon organics (Salehizadeh et al. 2020). Consequently, MES has drawn increasing attention in the fields of CO_2_ valorization and renewable energy stargate (Logan & Rabaey 2012). The concept of MES emerged around 2010 after some methanogens (Cheng & Logan 2007) and acetogens were found to be able to uptake electrons from the cathode for the production of methane and acetate, respectively. After that, a lot of work has been done to modify electrode materials, discover effective microorganisms, understand extracellular electron transfer (EET) mechanisms, and develop novel reactor configurations (Aryal et al. 2017; Krieg et al. 2014). The current density (i.e., productivity) of MES has improved a lot, but it is too low to bring this technology onto the market (Jourdin & Burdyny 2021; Prevoteau et al. 2020).

**Fig. 1:**
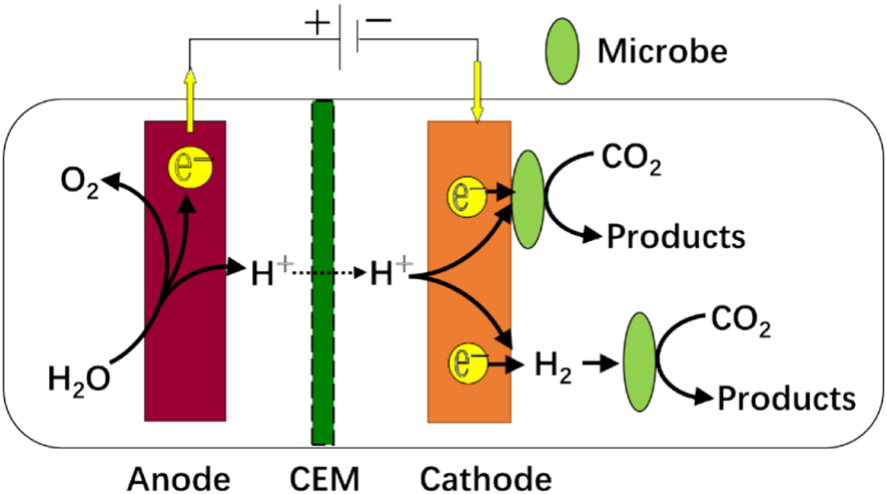
The schematic of MES reactors (CEM: cation exchange membrane)

Figure 1 shows the working principle of MES. In this system, renewable electricity is used to oxidize H_2_O to form O_2_, H^+^, and e^−^ in the anodic chamber, and then H^+^ and e^−^ are transported to the cathode chamber in which they are used by autotrophic microbes to fix CO_2_ for organic matter production. The conventional MES was driven by the biofilm on the cathode surface, because biofilm enabled high coulombic efficiency (CE), low energy input, and good biomass retention (Fruehauf et al. 2020). However, the current density of the biofilm-driven MES was limited by the activity of the biomass and the mass transfer efficiency. Currently, the current density of cathodic biofilms has reached the maximum values of electro-active biofilms (i.e., 10–100 A·m^− 2^) (Claassens et al. 2019). Thus, it is very challenging to further increase the current density of biofilm-driven MES, especially for its large-scale applications. Therefore, non-biofilm-driven MES has been proposed recently. This non-biofilm-driven MES used suspended biomass and hydrogen produced from the cathode for CO_2_ conversion, which was also named H_2_-mediated MES. Without the limitation of biofilm, high current density could be applied to the reactor for hydrogen production. However, the low solubility and poor mass transfer of H_2_ make it difficult to achieve high CE under high current density for this type of H_2_-mediated MES (Liu et al. 2016).

Previously, it has been reported that the enhancement of H_2_ mass transfer is an effective method to improve the performance of H_2_-mediated MES reactors. For example, Rodrigues et al. reported that adding perfluorocarbon nanoemulsion (a hydrogen carrier) into H_2_-mediated MES increased the productivity of acetate by 190% and obtained a CE of nearly 100% (Rodrigues et al. 2019). Our group also found that adding porous polyurethane (PPU) particles could promote the hydrogen uptake efficiency and acetate production rate in an H_2_-mediated MES reactor (Xue et al. 2022). Moreover, nanoparticles have also been applied to syngas fermentation and methane fermentation to improve the gas–liquid mass transfer (Kim et al. 2014; Zhu et al. 2008). Therefore, we hypothesized that introducing nanoparticles to H_2_-mediated MES may also enhance the hydrogen mass transfer, CE and acetate production rate. Among all reported nanoparticles, silica nanoparticles (SiO_2_ NPS) are biocompatible, dispersible in water, and commercially available at a relatively cheap price. Compared to perfluorocarbon nanoemulsion, SiO_2_ NPS can be easily separated from the fermentation broth for reuse. The PPU particles are on a millimeter scale, so that they can be easily maintained in the reactor, but the specific surface area of PPU is rather limited when compared to SiO_2_ NPS. Adding SiO_2_ NPS may further improve the hydrogen mass transfer in H_2_-mediated reactors. Thus, the goal of this study was to investigate whether the addition of SiO_2_ NPS could enhance the hydrogen mass transfer and the performance of an H_2_-mediated MES.

Our experimental results showed that the addition of SiO_2_ NPS in the MES system could significantly increase the H_2_ mass transfer rate and H_2_ solubility in water, thereby increasing the acetate production rate by 69.8% and the acetate titer by 56.9%. The average acetate production rate of the reactor with silica nanoparticles (2.14 g·L^− 1^·d^− 1^) was higher than those of the reported MES reactors. These results demonstrated adding SiO_2_ NPS is an efficient way to enhance the performance of H_2_-mediated MES reactors. The method reported here dramatically increases the current density and coulombic efficiency of H_2_-mediated MES reactors. It can not only be applied in H_2_-mediated MES reactors for acetate and methane production but also in other H_2_-dependent reactions, such as biologically catalyzed N_2_ fixation, CH_4_ functionalization and microbial protein production.

## Materials and methods

### Reactor design and construction

**Fig. 2:**
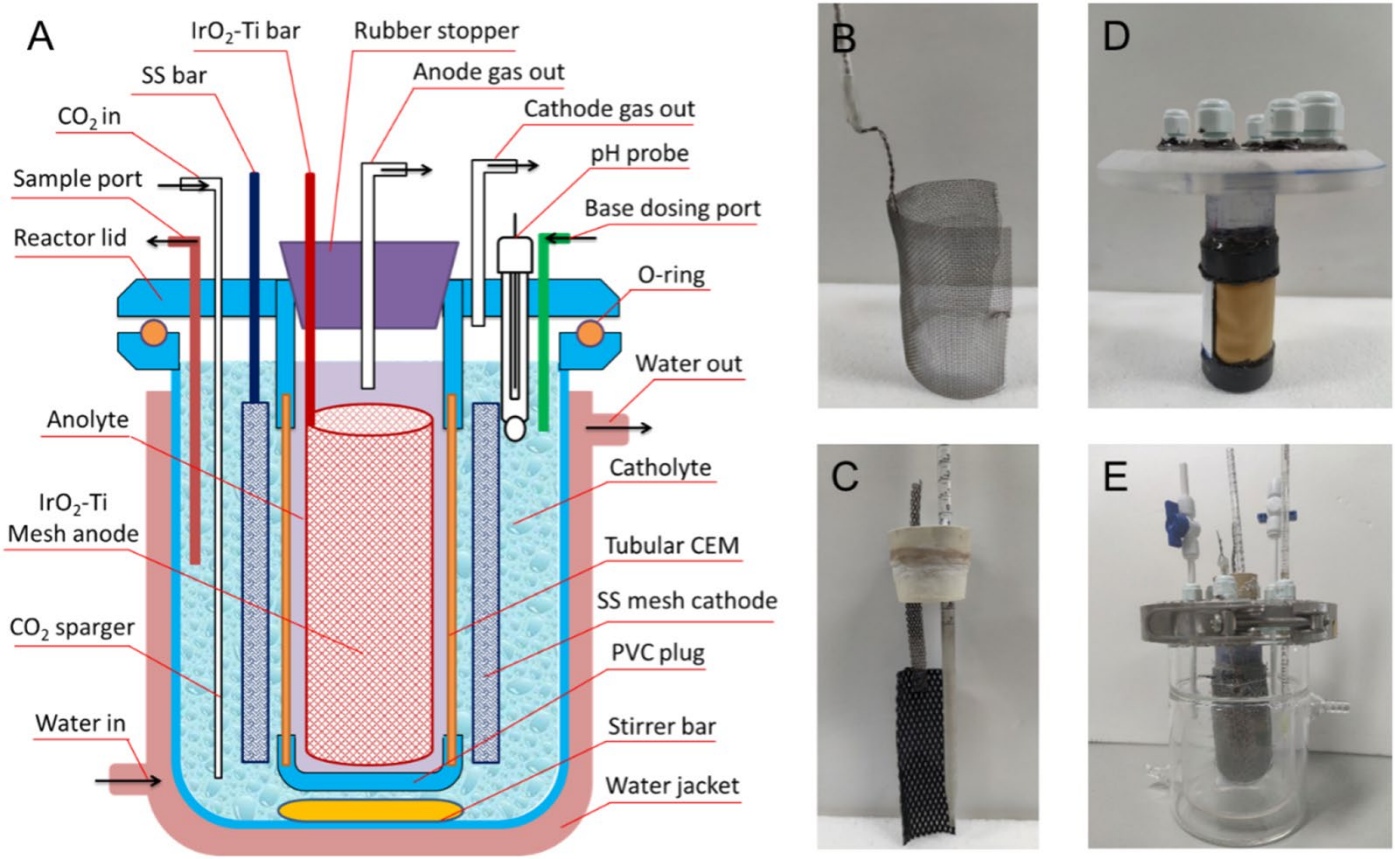
The schematic of the E-CSTR reactor (**A)** and photos of the reactor parts (**B**: the cathode; **C**: the anode; **D**: the anode membrane assembly; **E**: the jacketed mixing vessel)

An electrochemical continuous stirred-tank reactor (E-CSTR) was used for this study. The design of the E-CSTR is shown in Fig. 2. The reactor consisted of a tubular anode membrane assembly and a jacketed mixing vessel (outer diameter: 12.4 cm, inner diameter: 10.7 cm, height: 14.5 cm, maximum volume: 1 L). The anode membrane assembly consisted of a lid and a tubular membrane cell (diameter: 3.5 cm, height: 12.5 cm) that was placed in the middle of the lid. The bottom of the anode chamber was sealed by a PVC cap, while the top was plugged with a rubber stopper. The rubber stopper held the anode and an anode gas outlet tube. The size of the cation exchange membrane (CMI-7000, Membranes International Inc., USA) window on the anode membrane assembly was 42 cm^2^ (6 cm × 7 cm). There were six openings on the lid of the reactor, and they were used for the Stainless steel bar cathode current collector, CO_2_ inlet tube, base dosing tube, pH probe, cathode gas outlet tube, and liquid sample port. A stainless-steel mesh cylinder (length: 16 cm, height: 8 cm, thickness: 0.3 mm, Anguo Chengli Metal Co., Ltd., China) was used as the cathode. An IrO_2_-coated titanium mesh plate (width: 3.2 cm, height: 8 cm, Baoji Zhiming Special Metal Co., Ltd., China) was used as the anode. The reactor lid and the flange of the mixing vessel were sealed by an O-ring and a clamp.

The working volume of the anolyte and catholyte was 100 mL and 600 mL, respectively. The anolyte was 0.2 mol·L^− 1^ Na_2_SO_4_ solution, and its pH was adjusted to 2 with H_2_SO_4_. The catholyte was a modified M9 medium containing 6 g·L^−1^ Na_2_HPO_4_, 3 g·L^−1^ KH_2_PO_4_, 0.5 g·L^−1^ NH_4_Cl, 0.5 g·L^−1^ NaCl, 0.1 g·L^−1^ MgSO_4_·7H_2_O, 0.0146 g·L^−1 ^ CaCl_2_, 4 g·L^−1 ^ NaHCO_3_, 1 ml·L^−1^ trace element solution and 1 ml·L^−1^ vitamin solution. The compositions of the trace element solution and vitamin solution were provided in Table S1. The jacketed mixing vessel was connected to a recirculating water bath (DC-1006, Ningbo Scientz Biotechnology Co., Ltd., China) to control the reactor temperature. A magnetic stirrer was placed at the bottom of the vessel to control the agitation intensity of the catholyte. The anode and cathode were connected to the positive and negative terminals of the DC power supply (KA3005P, Korad Technology Co., Ltd., China), respectively. The pH of the catholyte was controlled with a pH controller (pH3.0-NI2-AC, Huzhou Tianze Biotechnology Co., Ltd., China) by automatically dosing 5 M NaOH. The reactor was equipped with a mass flow controller (D07-7, Beijing Sevenstar Flow Co., Ltd, China) to control the CO_2_ flow rate. A water displacement column (filled with 1200 mL 1 mM HCl) was connected to the reactor to collect the offgas for gas flow rate and composition measurement.

### Reactor startup and operational procedure

Two reactors were run in parallel, one as the control and the other one as the experimental reactor. The SiO_2_ NPS used in this study were commercial hydrophilic SiO_2_ NPS that was produced by the sol–gel method (average diameter 200 nm, Hebei Juli Metal Material Co., Ltd., China). The SiO_2_ NPS could easily disperse in water so they were used as purchased without any pretreatment.

In the control reactor, no SiO_2_ NPS were added, while in the experimental reactor 0.3wt% SiO_2_ NPS were added (1.8 g of SiO_2_ NPS were added into 600 mL of catholyte). The dosage of the SiO_2_ NPS (0.3wt%) was chosen based on literature regarding syngas fermentation enhancement by adding SiO_2_ NPS (Kim et al. 2014).

The reactor temperature was controlled at 35 ± 0.5 ℃ by the water bath, and the stirring rate of the magnetic stirrer was set at 650 rpm. The catholyte of the reactor was first flushed with N_2_ for 30 min to remove the dissolved oxygen. Then, the cathode chamber was inoculated with an enriched acetate-producing mixed culture that was dominated by *Acetobacterium*. The reactor was operated in a galvanostatic mode with a starting current of 0.25 A and then switched to 0.5 A. At 0.25 and 0.5A, the CO_2_ flow rate was controlled at 1.96 and 3.92 mL·min^− 1^, respectively, which resulted in an H_2_/CO_2_ molar ratio of 2:1. The pH of the catholyte was controlled at 7 using the pH controller.

Each day, 2 mL of catholyte was taken to measure the pH and VFAs, and the same amount of fresh catholyte was added. Occasionally, fresh anolyte was added to the anode chamber to compensate for the water loss. The reactors were operated in fed-batch mode. When the acetate concentration stopped increasing, 90% of the catholyte was replaced with a fresh medium to start a new batch. In the second batch, 0.3wt% SiO_2_ NPS were added to the experimental reactor.

### Analytical methods

#### Gas analysis

Gas samples were taken from the water displacement column to analyze the volume and composition of the unused gas. The gas composition, i.e., the concentrations of H_2_ and CO_2_, was analyzed by a compact gas chromatograph (GC, 7890B, Agilent). Details of the method could be found in our previous publication (Cai et al. 2022).

#### VFAs analysis

Liquid samples were taken from the reactor to analyze the concentration of acetate. The liquid samples were first extracted by diethyl ether and then injected into gas chromatography (GC-2010 Pro, AOC-201, Shimadzu, Japan) with an FID detector to analyze. Details of these methods could be found in our previous publication (Cai et al. 2022).

#### Hydrogen mass transfer coefficient measurement

The volumetric mass transfer coefficient (K_La_, h^− 1^) which describes the transfer resistance at the gas–liquid interface was tested by the dynamic-gasing method described in a previous article (Beckers et al. 2015). Briefly, the reactor was filled with M9 medium without inoculum. The temperature was kept at 35 ℃. The reactor was equipped with a dissolved hydrogen sensor (Clean, DH200, China) at the cover to measure and record dissolved H_2_ concentrations. The K_La_ of H_2_ in the reactor with SiO_2_ NPS and that without SiO_2_ NPS were measured under 0.5 A in abiotic conditions. For each experiment, the reactor was degassed with N_2_ to remove H_2_ before electrolysis. The values of the dissolved hydrogen sensor were recorded once a minute until reaching saturation.

#### Calculations

The k_La_ was calculated according to the adsorbing equation (Myung et al. 2016):

1 $$\frac{dC}{dt}={K}_{L}a\left({C}^{*}-C\right)$$

Here, ${C}^{*}$ is the saturated concentration of dissolved hydrogen (mg H_2_ L^− 1^), K_L_ is the mass transfer coefficient (cm·h^− 1^), and a is the gas/liquid interfacial area per volume of liquid (cm^2^·cm^− 3^).

The CE was calculated as described earlier (Liu et al. 2015), to reflect the productivity of the system:

2 $$CE = \frac{{\Delta {\text{C}}_{\text{HAc}} {\text{(mol L}}^{{ - 1}}) \times {\text{ V}}_{\text{solution}} {\text{(L) }} \times 8 \times {\text{ F (C mol}}^{{ - 1}} {)}}}{{\text{Overall charge (C)}}}{ } \times { } 100 \%$$

Here, ΔC_HAc_ (mol·L^–1^) is the change in the concentration of acetate during the experiment, V_solution_ is the total volume of the solution in the cathode chamber, and ‘Overall charge’ is the total electric charge passing through the cathode chamber and F is the Faraday’s constant.

## Results and discussion

### k_La_ determination and dissolved H_2_ concentration

**Fig. 3:**
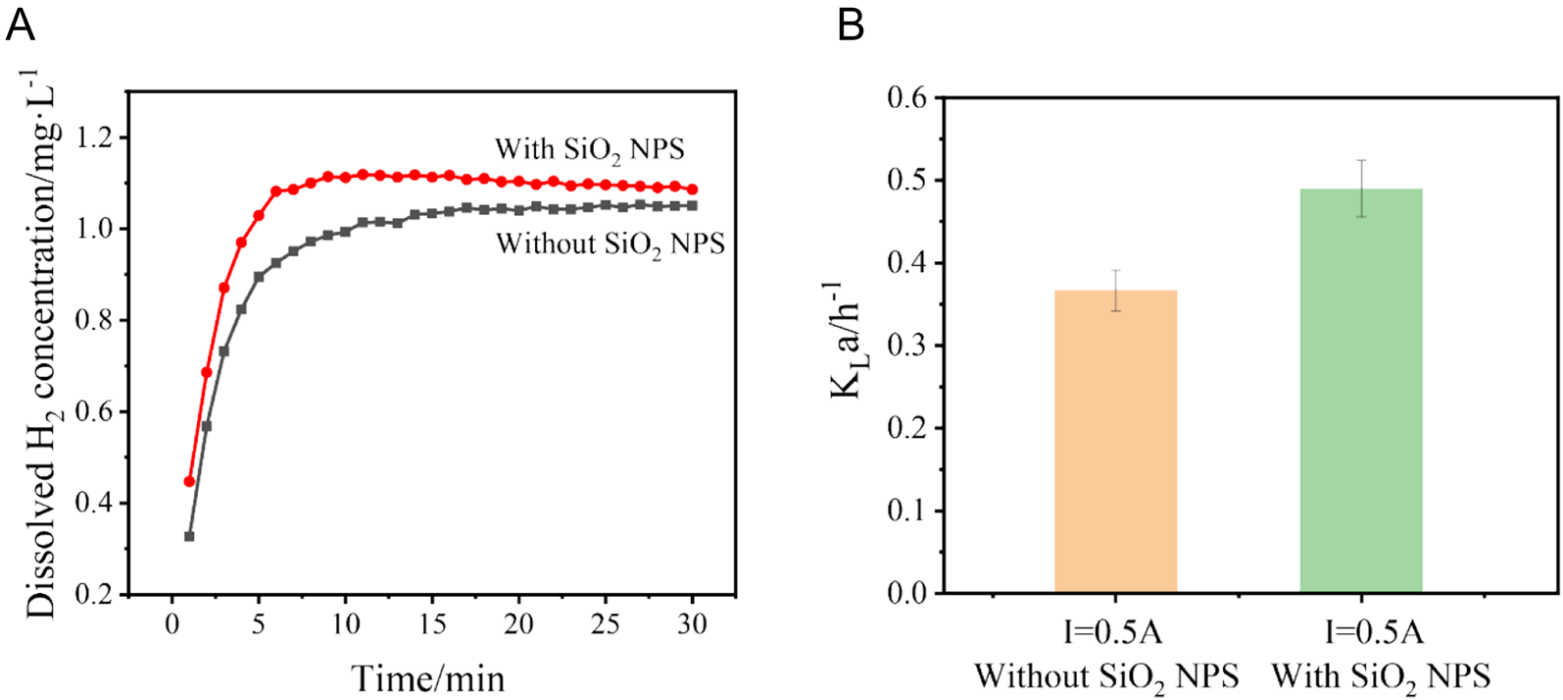
The dissolved H_2_ concentration curves (**A)** and the K_La_ of H_2_ (**B)** of reactors at 0.5 A under abiotic conditions

The K_La_ was measured under different conditions to determine the mass transfer rate of dissolved H_2_ at the gas–liquid interface (Fig. 3B). At 0.5 A, the K_La_ of the reactor added with SiO_2_ NPS reached 0.49 h^− 1^, which was 32.4% higher than that reached without SiO_2_ NPS (0.37 h^− 1^). The relation between the dissolved H_2_ concentration and the electrolysis time is shown in Fig. 3A. With the addition of SiO_2_ NPS, the saturated dissolved H_2_ concentration increased from 1.04 mg·L^− 1^ to 1.11 mg·L^− 1^ at 0.5 A. All these results confirmed that adding SiO_2_ NPS to the catholyte could improve the mass transfer of the dissolved H_2_ in the gas–liquid interface and increase the saturated dissolved H_2_ concentration of the reactor.

The kinetics of H_2_ oxidation by hydrogenase is rate-dependent for H_2_-induced CO_2_ reduction. As H_2_ has a limited solubility of 0.79 mM (Liu et al. 2016) in water at ambient conditions. Based on the information obtained from our experiments, the H_2_ K_La_ of the reactors could be increased by introducing SiO_2_ NPS. The improvement of the mass transfer coefficient by the interaction between nanoparticles and the gas–liquid interface could be explained by three theories: a shuttling or grazing effect, hydrodynamic influences at the gas–liquid interface, and changes in the specific gas–liquid interfacial area (Kim et al. 2014). The diameter of the nanoparticles was much smaller than that of the gas–liquid boundary layer, which is usually 5–25 μm. Therefore, it was likely that shuttling or grazing effects played an important role in improving gas–liquid mass transfer (Kim et al. 2014).

### Acetate production

**Fig. 4:**
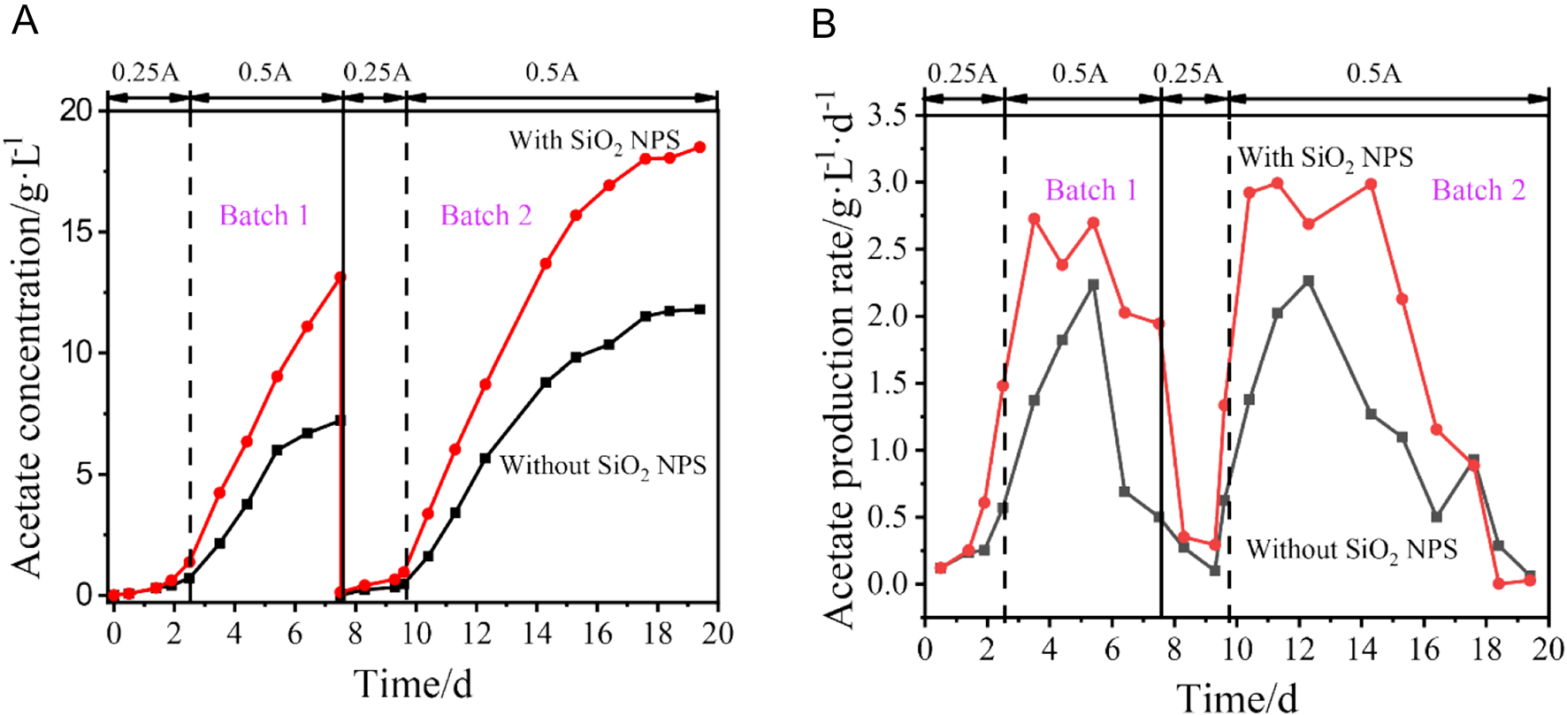
The acetate concentration (**A)** and acetate production rate (**B)** of the MES reactor in two batches

As shown in Fig. 4B, the acetate production rate was lower than 0.50 g·L^− 1^·d^− 1^ in the first 2 days, but it increased sharply in the coming days. When SiO_2_ NPS were present in the reactor, the acetate concentration increased from 0.29 g·L^− 1^ to 13.1 g·L^− 1^ (2–8 days), and the average acetate production rate reached 2.14 g·L^− 1^·d^− 1^ in batch 1. In contrast, the acetate production rate and the final acetate concentration of the experiment without SiO_2_ NPS were only 1.16 g·L^− 1^·d^− 1^ and 7.22 g·L^− 1^ in batch 1, respectively. The second batch displayed a comparable production profile, but the acetate titer outperformed the first batch. The maximum acetate concentration and average acetate production rate (10–18 days) of the experiments in batch 2 with (without) particles were 18.5 (11.8) g·L^− 1^ and 2.14 (1.26) g·L^− 1^·d^− 1^, respectively.

The performance of the second batch was superior to the first batch in terms of acetate titer due to the enrichment of more acetate-tolerating microorganisms during the long-term operation. In batch 2, the acetate production rate with nanoparticles was 1.7 times higher than that without nanoparticles at the stable increment stage (10–18 days) of acetate, and the titer of acetate increased by 56.9%. All these results suggested that the addition of SiO_2_ NPS could improve both the acetate production rate and titer of acetate by enhancing the mass transfer of the dissolved H_2_ at the gas–liquid interface and increasing the saturated dissolved H_2_ concentration in the reactor. This could be attributed to the fact that enhanced gas–liquid mass transfer would normally increase the amount of biomass in the reactor (Xue et al. 2022).

### Electron balance

**Fig. 5:**
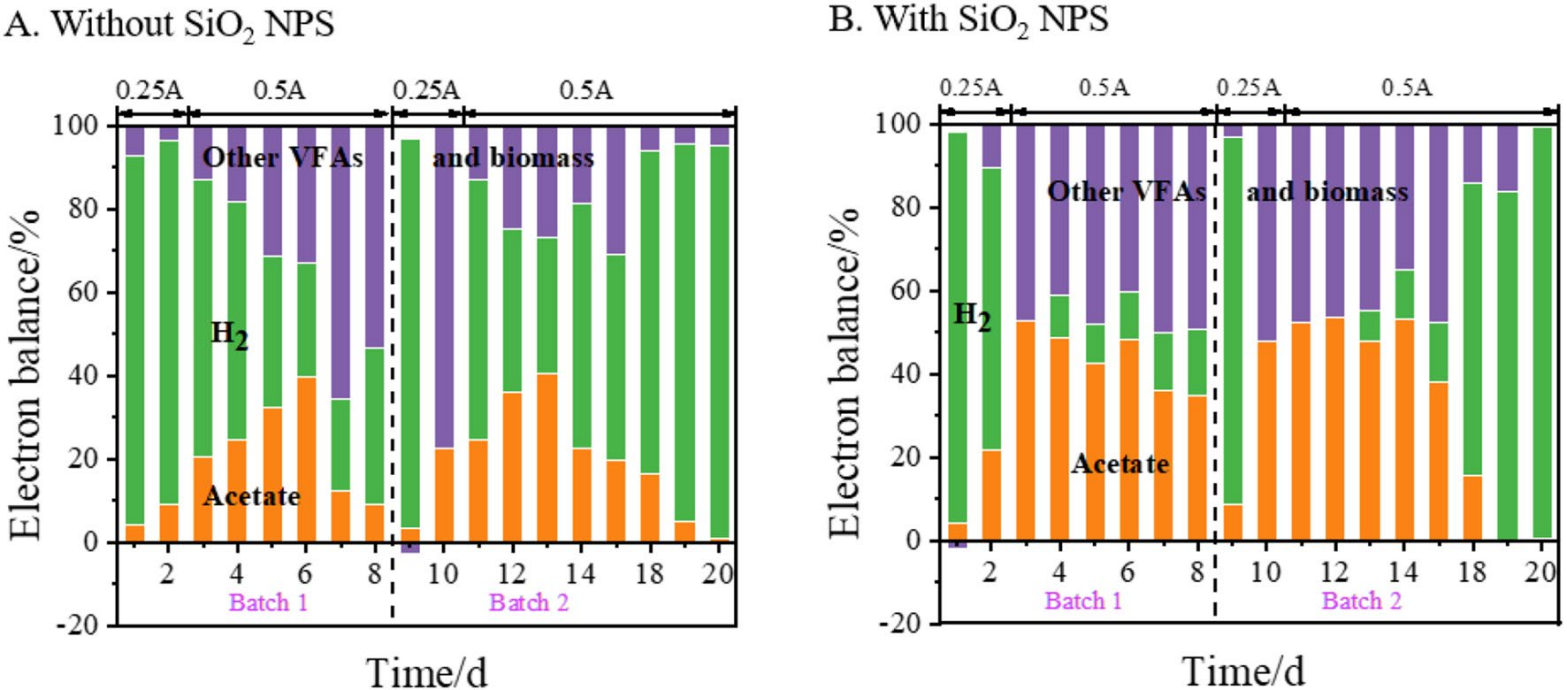
The electron balance of the MES reactor in two batches

Based on the VFAs concentration and the H_2_ left in the reactor, the electron balance was calculated. Most of the electrons from the cathode ended up in acetate and H_2_, while the rest could be attributed to other VFAs and biomass (Fig. 5). In general, the percentage of electrons that went to acetate increased at first and then decreased over time, whereas the percentage of electrons that ended in H_2_ decreased at first and then increased over time. In batch 1, the average percentages of electrons that went to the acetate of the reactor with and without SiO_2_ NPS were 36% and 19%, respectively. In the stable increment phase (10–18 days) of batch 2, the average percentage of electrons went to acetate, other VFAs and biomass, and H_2_ were 44%, 41%, and 15% with SiO_2_ NPS, while those without SiO_2_ NPS were 26%, 28%, and 46%, respectively. Therefore, the addition of SiO_2_ NPS could make more electrons go to acetate instead of H_2_.

In the stable increment phase, the reactor without SiO_2_ NPS reached its throughput bottleneck, because 50% of the electrical H_2_ was not utilized by the culture due to the limitation of H_2_ solubility in water. In contrast, the addition of SiO_2_ NPS can alleviate the throughput bottleneck by reducing the limitation and making almost all H_2_ be utilized by the culture.

### CO_2_ uptake efficiency

**Fig. 6:**
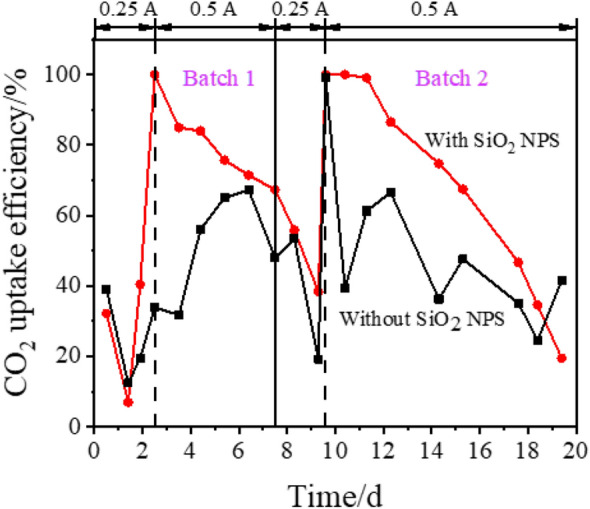
CO_2_ uptake efficiency of the MES reactor in two batches. At 0.25 A and 0.5 A, the CO_2_ flow rates were 1.96 mL·min^− 1^ and 3.92 mL·min^− 1^, respectively

The CO_2_ uptake efficiency (Fig. 6) was consistent with electron balance. To facilitate the acclimation of the microbial community, the reactor was operated under 0.25 A in the first two days. And the CO_2_ uptake efficiency of the reactor with SiO_2_ NPS reached 100%. Then, the applied current and the flow rate of CO_2_ were doubled. As shown in Fig. 6, the CO_2_ uptake efficiency with SiO_2_ NPS was over 60% except for some slight fluctuations, while the CO_2_ uptake efficiency without SiO_2_ NPS was between 30% and 70%. After that, the rapidly decreased CO_2_ uptake efficiency might be caused by product inhibition during the last two days of each batch. All these results indicated that the addition of SiO_2_ NPS could increase the CO_2_ uptake efficiency of the reactor.

In a broader context, SiO_2_ NPS may improve gas solubilities in water for a series of small non-polar gases, such as N_2_ and CH_4_, whose solubilities in an aqueous medium are also rate-limiting. We propose that SiO_2_ NPS are also potentially applicable for biologically catalyzed N_2_ fixation and CH_4_ functionalization (Rodrigues et al. 2019), two challenging processes in small molecule activation.

### Comparison to other MES reactors

The performance of this H_2_-mediated MES reactor was compared to other reported MES reactors in Table 1. Most studies were devoted to improving biofilm formation by developing effective cathodes. Apparently, the current density and acetate production rate of 3D porous cathodes were much higher than those of 2D cathodes in most reported MES reactors. It was simply because the 3D porous cathode could provide a higher specific surface area for the microorganisms and formed a thicker biofilm on the electrode. To our best knowledge, the acetate production rate of all reported MES reactors was less than 1.06 g·L^− 1^·d^− 1^, and the maximum acetate concentration was around 10 g·L^− 1^. By contrast, the acetate production rate and acetate titer of this reactor containing SiO_2_ NPS could reach 2.14 g·L^− 1^·d^− 1^ and 18.5 g·L^− 1^, respectively, which were about twice those of the reported reactors.

**Table 1:** Performance of most acetate-producing MES (Q _Surface_: Surface production rate; Q _Volumetric_: Volumetric production rate.)

Cathode material	Inoculum	V_catholyte_ (L)	J (A·m^−2^)	Q _Surface_ (g·m^−2^·d^−1^)	Q _Volumetric_ (g·L^−1^·d^−1^)	Titer (g·L^−1^)	CE (%)	Refs
Chitosan-coated carbon cloth	*Sporomusa* ovata	0.2	0.47	13.51	0.317	0.59	86	Zhang et al. 2013
Pr0.5BSCF-CF	Mixed culture	–	− 5.6	96	0.24	7.31	73	Tian et al. 2020
CF with fluidized GAC	Mixed culture	0.28	− 4	–	0.14	3.9	65	Dong et al. 2018
Mo_2_C-CF	Mixed culture	0.28	− 15	87.5	0.15	4.5	55	Huang et al. 2020
Graphite granules	Mixed culture	0.075	–	–	1.04	10.5	69	Marshall et al. 2013
rGO-CF	Mixed culture	0.28	− 4.9	68	0.17	7.1	77	Song et al. 2017
CNTs–RVC	Mixed culture	0.25	37	195	0.03	1.65	78	Jourdin et al. 2014
CF	Mixed culture	0.25	− 5	19	0.06	1.29	58	Patil et al. 2015
Carbon felt	Mixed culture	0.35	5	20.4	0.58	13.5	61	Gildemyn et al. 2015
VITO–CoRE	Mixed culture	0.5	0.069	46.7	0.14	4.97	45.5	Mohanakrishna et al. 2018
Graphene-carbon felt	*Sporomusa* ovata	0.25	− 0.23	62.4	0.124	1.88	83	Aryal et al. 2016
CNTs-coated Porous Ni hollow fiber	*Sporomusa ovata*	0.125	0.332	14.82	0.172	0.084	83	Bian et al. 2018
Carbon felt	Mixed culture	0.2	5	11.5	1.06	5.7	63	Arends et al. 2017
SS	Mixed culture	0.6	39	102	2.14	18.5	44	This work

These results could be partly attributed to the better mass transfer to suspended cells in the reactor. Compared to biofilm, the suspended cells had lower limitations caused by the substrate and product diffusion and electrode surface area, which could significantly improve the production rate of the bacteria. Furthermore, the addition of SiO_2_ NPS further increased the mass transfer rate of dissolved H_2_. However, nanoparticles could aggregate and gravitationally settle down in the stagnant flow region (dead zone) in the reactor. Moreover, the behavior of nanoparticles penetrating the cell cytoplasm may be toxic to the cell (Hwang et al. 2008). To obtain the best performance of acetate production, the optimum type and concentration of nanoparticles need to be further studied. In addition, many strategies, such as developing 3D structures and modifying functional groups on the surface of the nanoparticle could be utilized to increase the mass transfer rate and recyclability of nanoparticles. In the future, we could centrifuge the effluent to test whether it is possible to recover the silica nanoparticles for reuse, and we could also modify the silica nanoparticles into magnetic silica nanoparticles to reuse them.

## Conclusion

In summary, this work developed a novel strategy, i.e., adding SiO_2_ NPS, to improve the H_2_ solubility in the H_2_-mediated MES reactor. We found that the addition of SiO_2_ NPS in the MES system could significantly increase the H_2_ mass transfer rate and maximum saturated H_2_ solubility in water to accelerate the production rate and titer of acetate. With the addition of 0.3wt% SiO_2_ NPS, the H_2_ K_La_ of the reactor increased by 32.4% at 0.5 A. The titer of acetate in batch 2 of the reactor with SiO_2_ NPS (18.5 g·L^− 1^) was 56.9% higher than the reactor without SiO_2_ NPS (11.8 g·L^− 1^). In the stable increment phase, the average acetate production rate of the reactor with SiO_2_ NPS reached 2.14 g·L^− 1^·d^− 1^, which was much higher than other reports. This pioneering effort made with the addition of SiO_2_ NPS provided insights for the development of effective microbial electrosynthesis applications. In a broader context, the nanoparticle can also be utilized in other H_2_-dependent reactions, such as biologically catalyzed N_2_ fixation, CH_4_ functionalization and microbial protein production.

### Supplementary Information

**Additional file 1**: **Figure S1**. SEM of the SiO_2_ NPS. **Figure S2**. Dissolved H_2_ concentration curves (A) and K_La_ of H_2_ (B) of reactors at 0.25 A. **Figure S3**. H_2_ uptake efficiency (A) and CE (B) of the MES reactor in two batches. **Table S1**. Composition of trace element solution and vitamin solution.

## Data Availability

All data generated or analyzed during this study are included in this article.

## Abbreviations

MES: Microbial electrosynthesis
CEM: Cation exchange membrane
CE: Coulombic efficiency
K_La_: Volumetric mass transfer coefficient
EET: Extracellular electron transfer
SiO_2 _ NPS: Silica nanoparticles
PPU: Porous polyurethane
E-CSTR: Electrochemical continuous stirred-tank reactor
