# Supplementary material for: Enhancement of acetate production in hydrogen-mediated microbial electrosynthesis reactors by addition of silica nanoparticles
Source: Bioresour Bioprocess. 2023 Jan 20;10(1):3. doi: 10.1186/s40643-023-00627-6 (PMC10992923; doi:10.1186/s40643-023-00627-6)
Supplement: Supplementary file 1 — Additional file 1: Figure S1. SEM of the SiO2 NPS. Figure S2. Dissolved H2 concentration curves (A) and KLa of H2 (B) of reactors at 0.25 A. Figure S3. H2 uptake efficiency (A) and CE (B) of the MES reactor in two batches. Table S1. Composition of trace element solution and vitamin solution. [file 40643_2023_627_MOESM1_ESM.docx]

**Supporting Information**

Enhancement of acetate production in hydrogen-mediated microbial electrosynthesis reactors by addition of silica nanoparticles

Zeyan Pan ^1^, Zhuangzhuang Liu^1,2^ , Xiaona Hu^3^, Kai Cui^1^, Wenfang Cai^1^, Kun Guo^1*^

*^1^ School of Chemical Engineering and Technology, Xi’an Jiaotong University, Xi’an 710049, China*

*^2^ College of Veterinary Medicine, Northwest A&F University, Yangling, 712100, China*

*^3^ School of Ecology and Environment, Zhengzhou University, Zhengzhou 450001, China*

*Corresponding Authors:

*[kun.guo@xjtu.edu.cn](file:///C:\\Users\\Lenovo\\Desktop\\kun.guo@xjtu.edu.cn)*


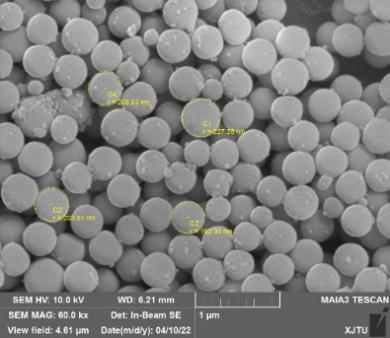

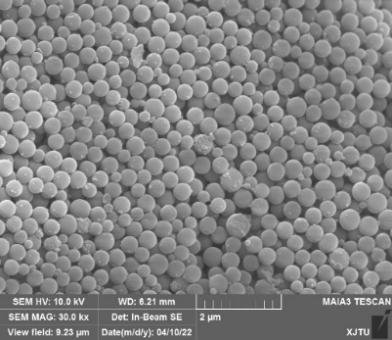

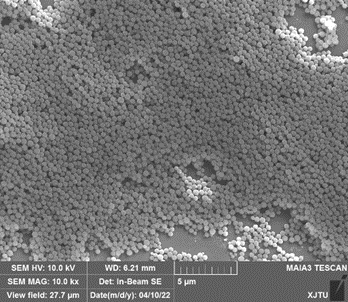


Figure S1**.** SEM of the SiO_2_ NPS


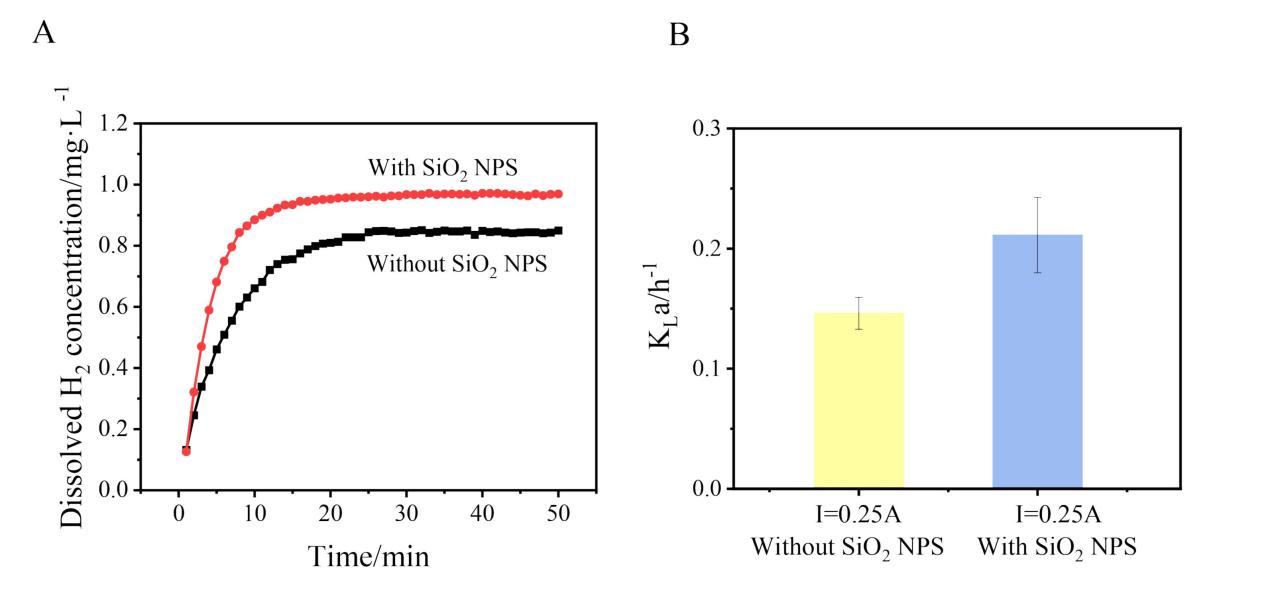


Figure S2**.** The dissolved H_2_ concentration curves (A) and the K_La_ of H_2_ (B) of reactors at 0.25 A


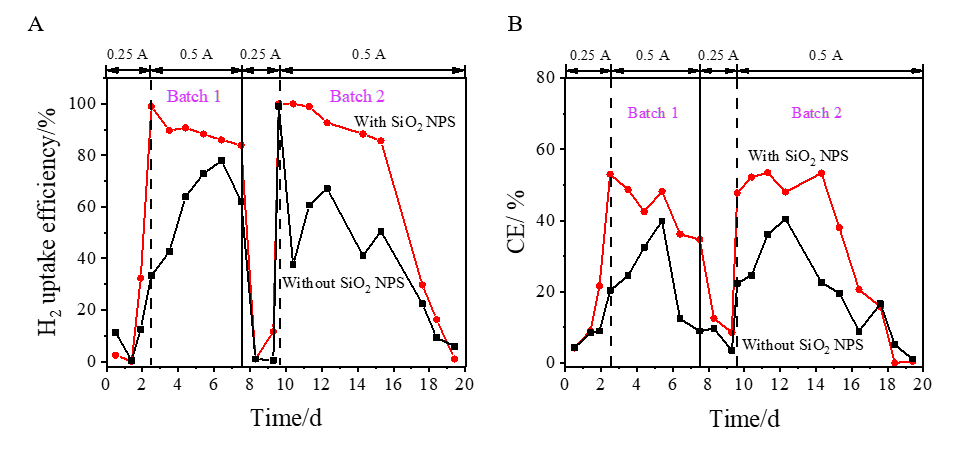


Figure S3**.** The H_2_ uptake efficiency (A) and CE (B) of MES reactor in two batches.

Table S1. Composition of trace element solution and vitamin solution

| **Trace element solution** | | **Vitamin solution** | |
| --- | --- | --- | --- |
| Chemical | g/L | Chemical | g/L |
| KI | 0.18 | Biotin | 0.002 |
| H_3_BO_3_ | 0.15 | Folic acid | 0.002 |
| CuSO_4_·5H_2_O | 0.03 | Vitamin B12 | 0.0001 |
| FeCl_3_·6H_2_O | 1.5 | Riboflavin | 0.05 |
| MnCl_2_·4H_2_O | 0.12 | Thiamine | 0.05 |
| CoCl_2_·6H_2_O | 0.15 | Nicotinic acid | 0.05 |
| ZnSO_4_·7H_2_O | 0.12 | Pantothenic acid | 0.05 |
| Na_2_MoO_4_·2H_2_O | 0.06 | Pyridoxine-HCl | 0.01 |
| EDTA (acid form) | 10 | p-Aminobenzoic acid | 0.05 |
| NaOH | Adjust pH to 7 | Thioctic acid | 0.05 |
| NiCl_2_·6H_2_O | 0.023 | \|  \|  \| \| --- \| --- \| |  |
